# Primary, Indifferent, and Secondary Effects

**Published:** 1895-07

**Authors:** A. McNeil

**Affiliations:** San Francisco, Cal.


### PRIMARY, INDIFFERENT, AND SECONDARY EFFECTS.

A. McNeil, M. D., San Francisco, Cal.

The following contains a point on the question of potency that I have never seen or heard, and that I think is one that should not be overlooked. It is from *Die Homëoptus*, by Prof. Gustav Jaeger, page 26: "In a certain dose the size of which depends on the effects of the drug—*i. e.*, in some it is large, in others very small—it produces no perceptible changes in the physiological functions, viz.: that which we may call the *indifferent* or neutral dose, on both sides of this indifferent dose there lies what I have called the quantitative antagonisms of drug action. If we give more than an indifferent dose there appears after a transitory exciting effect (acceleration of certain vital processes), a decided depressed action (a slowing of certain vital processes), and a group of phenomena which we designate as poisonous arise, and these are the more intense the larger the dose or the more concentrated it is until it becomes a lethal one which is a total paralysis. On the other hand, if we administer the drug in a dose smaller or less concentrated than the indifferent one then the physiological effect is a pure excitatory one compared with the primary effect which the drug produces in a poisonous dose, and as with the increase of the concentration the depressive effect increases, so increases on the other hand with the decrease of the dose—*i. e.*, with the increase of the dilution, the excitory effect."

